# Costs and benefits of admixture between foreign genotypes and local populations in the field

**DOI:** 10.1002/ece3.3946

**Published:** 2018-03-05

**Authors:** Jun Shi, Jasmin Joshi, Katja Tielbörger, Koen J. F. Verhoeven, Mirka Macel

**Affiliations:** ^1^ Institute of Evolution and Ecology Plant Ecology Group University of Tübingen Tübingen Germany; ^2^ Ningbo Academy of Agricultural Sciences Ningbo China; ^3^ Biodiversity Research/Systematic Botany Institute of Biochemistry and Biology University of Potsdam Potsdam Germany; ^4^ Berlin‐Brandenburg Institute of Advanced Biodiversity Research (BBIB) Institute of Biology Freie Universität Berlin Berlin Germany; ^5^ Department of Terrestrial Ecology Netherlands Institute of Ecology (NIOO‐KNAW) Wageningen the Netherlands; ^6^ Molecular Interaction Ecology Department of Plant Science Radboud University Nijmegen Nijmegen the Netherlands; ^7^ Plant Ecology and Phytochemistry Leiden Institute of Biology Leiden the Netherlands

**Keywords:** heterosis, inbreeding depression, local adaptation, *Lythrum salicaria*, outbreeding depression

## Abstract

Admixture is the hybridization between populations within one species. It can increase plant fitness and population viability by alleviating inbreeding depression and increasing genetic diversity. However, populations are often adapted to their local environments and admixture with distant populations could break down local adaptation by diluting the locally adapted genomes. Thus, admixed genotypes might be selected against and be outcompeted by locally adapted genotypes in the local environments. To investigate the costs and benefits of admixture, we compared the performance of admixed and within‐population F1 and F2 generations of the European plant *Lythrum salicaria* in a reciprocal transplant experiment at three European field sites over a 2‐year period. Despite strong differences between site and plant populations for most of the measured traits, including herbivory, we found limited evidence for local adaptation. The effects of admixture depended on experimental site and plant population, and were positive for some traits. Plant growth and fruit production of some populations increased in admixed offspring and this was strongest with larger parental distances. These effects were only detected in two of our three sites. Our results show that, in the absence of local adaptation, admixture may boost plant performance, and that this is particularly apparent in stressful environments. We suggest that admixture between foreign and local genotypes can potentially be considered in nature conservation to restore populations and/or increase population viability, especially in small inbred or maladapted populations.

## 1 INTRODUCTION

Population admixture (hereafter: admixture) is a genetic process of hybridization between previously isolated populations of one species (Ellstrand & Schierenbeck, 2000). Admixture might, for example, happen via translocation of seeds or removal of isolation barriers. In this way, it may be an important process in invasion biology where genotypes from various origins are introduced into a new area (Lavergne & Molofsky, 2007). It is also a concern in restoration ecology, where the origin of seed sources used for restoration is still under debate (Bucharova et al., 2017; Gellie, Breed, Thurgate, Kennedy, & Lowe, 2016) and where both positive and negative effects of admixture may occur. Admixture can be beneficial by leading to heterosis and lifting inbreeding depression (Escobar, Nicot, & David, 2008). Mildly deleterious alleles that are expressed in homozygotes could be masked through admixture in the F1 hybrid offspring (Hufford & Mazer, 2003). This may be particularly beneficial for small isolated populations that are prone to suffer from inbreeding depression (Hedrick & Kalinowski, 2000). Admixture can also lead to heterosis via a general fitness advantage of heterozygotes (overdominance; Charlesworth & Willis, 2009). Furthermore, admixture can increase the genetic variation of a population or lead to novel adaptive genotypes (Verhoeven, Macel, Wolfe, & Biere, 2011).

While heterosis can be a benefit of admixture, the introduction of novel genotypes can also cause outbreeding depression, that is, an increase in maladapted genotypes, which is the main potential fitness cost of admixture. A mechanism for outbreeding depression is “dilution” of locally adapted genomes (Hufford & Mazer, 2003). Species can evolve local adaptation due to divergent selection by the local environments (Leimu & Fischer, 2008; Linhart & Grant, 1996). Factors that can play a role in local adaptation can be abiotic, for example, climate and soil chemistry, or biotic such as herbivores, pathogens, and mutualists (Cremieux et al., 2008; Macel et al., 2007). Admixture would dilute locally adapted genomes in the admixed offspring (Keller, Kollmann, & Edwards, 2000). Selection would therefore put constraints on the dilution of locally adapted gene pools through substantial fitness loss of the admixed offspring relative to either parent (Angert, Bradshaw, & Schemske, 2008). Consequently, this local adaptation might contribute to isolation of populations by selection against nonlocal genotypes in a population thus reducing admixture and may enhance inbreeding (Nosil, Vines, & Funk, 2005; Verhoeven et al., 2011).

On a genetic level, if the parents are genetically very distant, there may be a disruption of co‐adapted gene complexes via recombination (Lynch, 1991). This may lead to reduced viability or fertility in case of serious genetic incompatibilities, also known as “hybrid breakdown” (Rius & Darling, 2014). As recombination of a co‐adapted gene complex first occurs in the second hybrid generation, hybrid breakdown might only occur in subsequent F2 or later generations (Fenster & Galloway, 2000; Hathaway, Andersson, & Prentice, 2009; Hufford & Mazer, 2003).

Thus, the intrinsic genetic benefits of admixture through heterosis and/or from increased adaptive potential in admixed progenies are intertwined with the environmentally dependent costs of admixture from the dilution of locally adapted genomes in the local habitat and hybrid breakdown in later generations (Keller & Taylor, 2010; van Kleunen, Rockle, & Stift, 2015; Lavergne & Molofsky, 2007; Rius & Darling, 2014).

To our knowledge, until now only a few studies have looked at the balance between the costs and benefits of admixture in F1 and F2 offspring of plants in the field (Cremieux, Bischoff, Muller‐Scharer, & Steinger, 2010; Pickup, Field, Rowell, & Young, 2013; Verhoeven, Vanhala, Biere, Nevo, & Van Damme, 2004). Furthermore, most of those studies were limited to one field site with one local population instead of using multiple sites and their local populations, which makes it difficult to generalize the results for a given species.

Here, we tested the performance of F1 and F2 offspring of within and between population crosses of the invasive plant *Lythrum salicaria* of three European regions in its native range in the field. We used a reciprocal transplant approach to test for local adaptation and the effect of admixture on plant performance in the field. Performance of local versus foreign genotypes within each site as well as the plant performance at home versus the plant performance away can indicate local adaptation (Joshi et al., 2001; Kawecki & Ebert, 2004). We had three common gardens across Western Europe in close proximity of our seed origins and used local soil inocula from the home population at each common garden because plants may be adapted to local soil biota. If admixture is decreasing the performance of the local population due to the dilution of locally adapted genomes, we would expect a negative effect of admixture at the home site but not at the foreign sites. We measured plant growth and reproduction, and herbivory in order to answer the following questions: (1) Is there local adaptation in native *L. salicaria*? If *L. salicaria* shows local adaptation, then (2) does admixture negatively affect plant performance of locally adapted populations in their local environments (dilution of local adaptation) but not in other environments? And if there is no local adaptation, then (3a) does admixture enhance plant performance (heterosis)? Or (3b) does admixture decrease plant performance (hybrid breakdown)?

## 2 METHODS

### 2.1 Study species

*Lythrum salicaria* L. (Purple Loosestrife; Lythraceae) is an erect, wetland herbaceous perennial plant (Thompson, Stuckey, & Thompson, 1987). It is heterostylous and each plant produces one of three morph‐specific patterns: long‐, mid‐ or short‐styled morph (Waites & Agren, 2004). The trimorphic system in *L. salicaria* avoids self‐pollination (Colautti, White, & Barrett, 2010; Knuth, 1898) as legitimate pollination requires the deposition of pollen on the stigma from anthers of equivalent height, which are found only between different flower style lengths (Eckert, Manicacci, & Barrett, 1996; Oneil, 1992; Waites & Agren, 2004).

### 2.2 Plant material and experimental crosses

#### 2.2.1 *Lythrum salicaria* seed collection

In September 2012, seeds of native European *L. salicaria* were collected from three populations in each of three regions, Tübingen, Potsdam, and Wageningen, respectively (nine populations in total; Table 1). The geographical distances between populations within each region ranged from 3 to 15 km, and the three regions were approximately 600 km apart from each other. The collected seeds were stored at 4°C. The geographical position of the collection sites, altitude, and annual temperature is illustrated in Table 1.

**Table 1:** Geographical position of *Lythrum salicaria* populations of which seeds were collected in 2012

Region	Population	Latitude (N)	Longitude (E)	Altitude (m)	Annual temperature (°C)	January temperature (°C)	July temperature (°C)	Annual precipitation (mm)
Tübingen (Germany)	Hagelloch	48°32′36″	09°00′58″	492	11.0^a^	3.0^a^	20.7^a^	592.6^a^
	Unterjesingen	48°31′06″	08°58′54″	347				
	**Reusten**	48°33′13″	08°56′17″	413				
Potsdam (Germany)	Geltow	52°22′10″	12°57′20″	30	10.8^a^	1.7^a^	20.4^a^	553.8^a^
	Ferch	52°19′56″	12°55′30″	62				
	**Golmer Luch**	52°23′48″	12°56′56″	30				
Wageningen (The Netherlands)	Ewijk	51°52′39″	05°45′04″	10	11.3^b^	4.9^b^	19.1^b^	1002.0^c^
	Nijmegen	51°50′57″	05°53′30″	11				
	**Wageningen**	51°58′43″	05°40′42″	13				

In bold: focal local populations in the transplant experiment, used as seed parental populations. The other populations functioned only as pollen donors in the crosses used for this experiment.

a http://www.wetterkontor.de/(2014–2015, temperature and precipitation of Potsdam and Tübingen (Stuttgart) regions

b https://weerstatistieken.nl/(2014–2015, temperature of Wageningen (De Bilt) region)

c http://historie.neerslagkaart.nl//(2014–2015, precipitation of Wageningen region based on source KNMI)

#### 2.2.2 F1 generation of *Lythrum salicaria*

In October 2012, seeds from 12 to 15 mother plants per population of all nine populations were sown in petri dishes with water in a greenhouse with 16‐hr light, 8‐hour dark, and a constant 20°C. Two weeks later, one seedling per mother plant was transplanted into 1.5 L pots filled with steamed commercial potting soil. In total, 116 seedlings were planted in the same greenhouse as used for the germination with the same conditions. Around 50 days after transplantation, three types of pollinations were made: (1) between plants within a single population (intrapopulation crosses), (2) between plants from different populations in the same region (interpopulation crosses), and (3) between plants from different regions (interregional crosses). For each of the nine populations, the 12–15 plants grown per population were used both as seed parent and as pollen donor for all cross types. Due to incompatibility within the style morphs (tristylous mating system; Eckert et al., 1996), not all seed parents could be used for all cross types. In the end, of each population, there were 4–7 seed parents that were used for all three cross types, receiving pollen from within‐population, within‐region, and between‐region pollen donors that were randomly chosen from available plants. The remaining seed parents only received pollen from one or two cross types. Cross type × population identifiers were defined by the name of each seed parent (not pollen donor). Thus, for example, “Potsdam interregional cross” denotes seeds from a Potsdam plant that was crossed with one of the other European populations. In April 2013, the seed capsules from each plant were harvested and stored at 4°C.

#### 2.2.3 F2 generation of *Lythrum salicaria*

In August 2013, seeds from 10 mothers of the F1 generation per focal population and cross type were sown in petri dishes. The growing conditions were the same as that in “F1 generation of *L. salicaria*.” The F2 generation was made by only crossing the F1 plants within each population and cross type. For the interpopulation and interregional crosses, this meant that some pollen donor populations for the F2 where not identical to the pollen donors of the F1 (e.g., Tübingen Reusten × Wageningen Ewijk could be crossed with Tübingen Reusten × Potsdam Golm). In February 2014, the seed capsules from each plant were harvested and also stored at 4°C.

### 2.3 Reciprocal transplant common garden experiment

In June 2014, three common gardens in each region, Tübingen (48°32′N, 09°02′E), Potsdam (52°24′N, 13°01′E), and Wageningen (51°59′N, 05°40′E), were prepared for our reciprocal transplant experiment. We selected three focal local populations (seed parents) for the experiment, one of each region (Table 1). The distance from the common garden in each region to the focal local *L. salicaria* population was around 2.5 km in Wageningen, 6 km in Potsdam, and 8.5 km in Tübingen. The average annual/monthly temperature and precipitation of each region of 2014 and 2015 are provided in Table 1 and Figure S1.

In each common garden, F1 and F2 offspring of the intrapopulation crosses and the interregional crosses of the same three focal populations were planted (one local focal population and two foreign focal populations per site). Only for the local focal population of each common garden, also the offspring of the interpopulation crosses were included to test the effect of admixture with close‐by versus distant parents on the performance of the local population in the field.

At each site, 10 replicates of each population × cross type × generation were planted, 140 plants in total: one focal local population × two generations (F1 and F2) × three cross types × 10 replicates + two foreign populations × two generations (F1 and F2) × two cross types × 10 replicates.

At each common garden, seeds from 10 families per population per generation in each cross type were sown in seed trays in a greenhouse with natural light and humidity and a minimum temperature of 18°C at the end of May 2014. Three weeks later, one randomly selected seedling from each family was transplanted into a 15 L pot in the respective common garden. Bulk field soils were collected from fields nearby the common gardens in each region and steam sterilized. Additionally, in each local focal population, 140 L of local soil was collected. Pots were filled with 14 L steam‐sterilized bulk field soil and 1 L local nonsteamed field soil to inoculate the bulk soil with the local soil biota. In each experimental garden, the pots thus contained a different bulk background field soil inoculated with soil from the focal local population. Pots were placed on large dishes (diameter 28 cm). In the Tübingen and Potsdam common gardens, water was supplied manually when needed, keeping the soils wet. In the Wageningen common garden, plants were watered through an automatic watering system. At each common garden site, the pots were placed in a completely randomized design.

There were three rounds of censuses, one at the end of the first growing season, one in the middle of the second growing season, and one final census at the end of the second growing season. Biomass, plant height, and fruit production were used as a proxy for fitness of this perennial plant. Plant height was recorded at all three censuses as the vertical length (cm) from the surface of soil to the top of the plant. Plant height was highly correlated with total biomass (Spearman's correlation at final harvest: *R*_s_ = 0.70, *p* < .0001, *N* = 418) and thus a good indicator for biomass throughout the experiment. Biomass itself was only harvested at the final census; all aboveground biomass was harvested, dried for 3 days at 65°C and weighed. At the end of each growing season, the length of each inflorescence stalk was measured and summed for total inflorescence length (cm). We randomly selected two 10‐cm‐length sections on inflorescence stalks on each plant to calculate average fruit number per 10 cm. These data were then used to estimate total fruit production per plant per year, and fruits year 1 and fruits year 2 were summed for total fruit number. Time to first flowering can be an indication for adaptive latitudinal clines in plants, where plant from higher latitudes tend to flower earlier (Montague, Barrett, & Eckert, 2008). In the second growing season, the time to first flower, from 1st May to the day of first flowering in each plant, was therefore also recorded. Adaptation to the local herbivore community may play a role in local adaptation (Cremieux et al., 2008). At the end of the first growing season and in the middle of the second growing season, herbivore damage to leaves was measured. In each plant, eight leaves from the main stem were randomly selected to score how many leaves showed signs of damaged by herbivores.

### 2.4 Statistical analyses

Statistical analyses were conducted using SPSS version 21. The effect of plant population, experimental site, cross type, and plant generation on all plant performance traits were analyzed with a multivariate analysis of variance (MANOVA), because of the interdependency of the measured traits. Site, population, cross type, and generation were set as fixed factors and their full factorial interactions were added to the model. Plant total biomass was log^10^ transformed, and total number of fruits were square root transformed to meet the assumptions of normal distribution and homoscedasticity. Herbivory (the proportion of the number of leaves attacked by herbivores/eight randomly selected leaves) was analyzed with generalized linear models with binomial distribution and a logit function. Differences between the groups were analyzed with post hoc Tukey's tests where relevant. We tested for local adaptation within the intrapopulation crosses (no admixture) using linear contrasts within the above model. Local versus foreign contrasts between the performances of the local population versus the two foreign populations were tested for each experimental site separately (Kawecki & Ebert, 2004). *p*‐values were adjusted for multiple comparisons using false discovery rates (FDR).

## 3 RESULTS

### 3.1 Overall effect of site and population on plant performance

**Figure 1:**
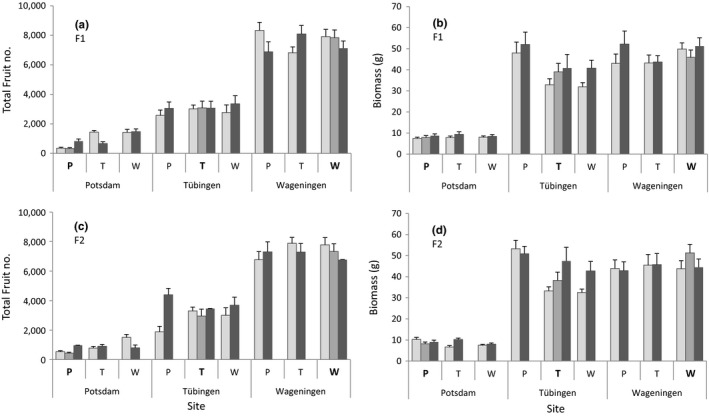
Estimated total fruit number (a) and total biomass (b) of F1 offspring of different crosses of focal *Lythrum salicaria* populations of three regions at common gardens in each region, and fruit number (c) and biomass (d) of the F2 offspring. P indicates Potsdam‐Golmer Luch as focal population (seed donor), T indicates Tübingen‐Reusten as focal population, W indicates Wageningen–Wageningen as focal population, in bold are the home populations. Light gray bars indicate intrapopulation crosses (C1), middle gray bars indicate the interpopulation crosses with a region (C2), and dark gray bars indicate the interregion crosses (C3). Error bars indicate standard errors. *N* = 10 per site × population × cross type × generation

**Figure 2:**
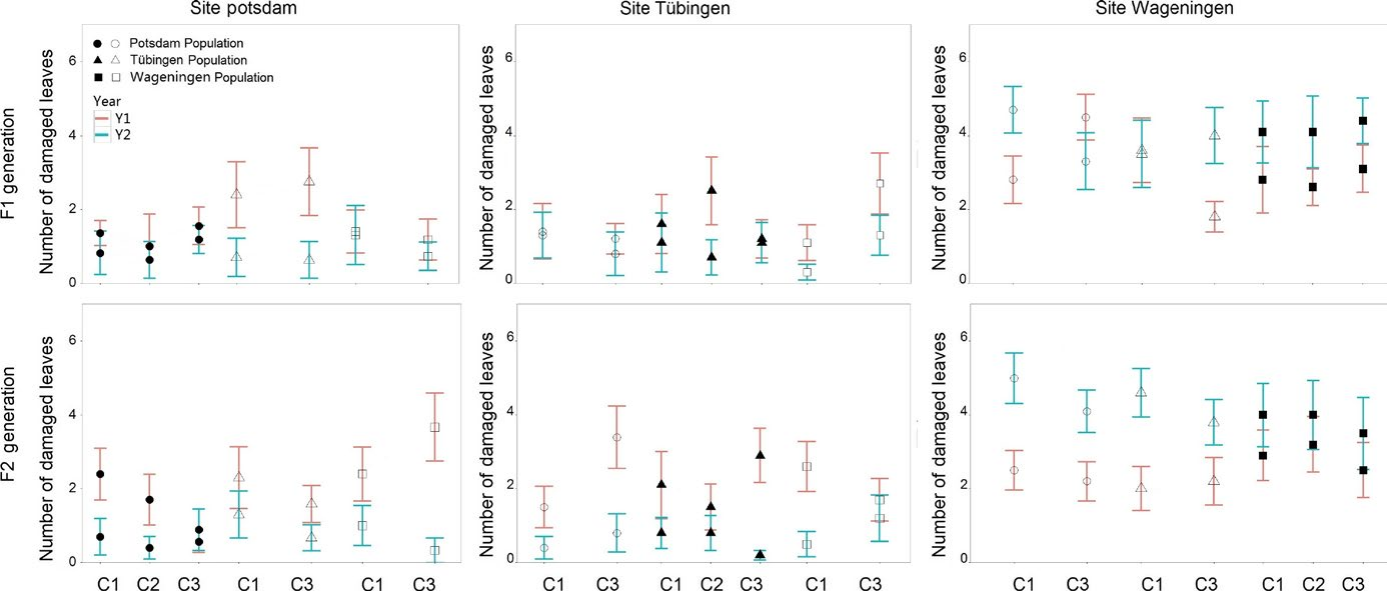
Mean and standard error of number of leaves damaged by herbivores of F1 and F2 offspring of different crosses of focal *Lythrum salicaria* populations of three regions at common gardens in each region. Pink line shows the results of the first year and green line of the second year. The solid symbols indicate the local population in their home site, and open symbols indicate the foreign populations. C1, Intrapopulation crosses; C2, Interpopulation crosses; C3, Interregional crosses. *N* = 10 per site × population × cross type × generation

Survival was high in our pot experiment. All 140 plants survived until the end of the experiment at the Wageningen site, one plant died in Tübingen and one plant died in Potsdam. While survival was equally high among the sites, the effect of site on plant growth and reproduction was significant (Table 3). In general, plant performance was lowest at the Potsdam common garden and highest at Wageningen common gardens (Figure 1, Table S1; Post hoc Tukey's tests *p *<* *.0001). Herbivory was highest at the Wageningen site, especially in the second year of the experiment (Figure 2; Post hoc Tukey's test *p *<* *.0001). The effect of plant population origin was significant for biomass and fruit number, and near significant for the other traits (Table 3). Overall, the Wageningen *Lythrum* population produced more fruits than the Tübingen and Potsdam populations, and fruit production of the Potsdam population was lowest (post hoc Tukey's tests *p *<* *.0001). The Potsdam population also had the overall lowest biomass compared to the other two populations (*p *≤* *.002). The Wageningen population flowered 1–2 days earlier than the Potsdam population (Table 2, *p* = .001), time to flowering of the Tübingen population was not significantly different from the other two populations. However, the effect of plant population also depended on site as indicated by the significant site by population interaction for some of the traits (Table 3).

**Table 2:** Mean and standard error of the time to first flowering (the number of days from 1st May to the time of first flowering) of each cross type and each population in each experimental site. Data of F1 and F2 generations per population x cross type were combined, and interpopulation crosses were only included for the local populations

		Site		
Population	Cross type	Potsdam	Tübingen	Wageningen
Potsdam	Intrapopulation	65 ± 1	48 ± 1	56 ± 1
	Interpopulation	65 ± 2	–	–
	Interregion	60 ± 1	47 ± 2	57 ± 1
Tübingen	Intrapopulation	61 ± 1	47 ± 1	56 ± 1
	Interpopulation	–	52 ± 1	–
	Interregion	62 ± 2	49 ± 1	56 ± 1
Wageningen	Intrapopulation	59 ± 1	44 ± 1	55 ± 1
	Interpopulation	–	–	57 ± 2
	Interregion	60 ± 1	47 ± 1	54 ± 1

**Table 3:** Effects of site, plant population, cross types, and generation on plant performance and herbivory. Plant performance data were analyzed by full factorial MANOVA with site, population, cross type, and generation as fixed factors. Table entries of above traits are *F* values. Herbivory was analyzed separately by generalized linear models with binomial distribution. *N* = 419. Height1, Height2, and Height3 indicate stem height at the end of the first growing season, and at the middle and end of the second growing season, respectively. Fruit no. is the estimated total number of fruits produced during the experiment. Herbivory1 is the herbivory measurement in year 1, Herbivory2 of year 2

Factor	*df*	Height1	Height2	Height3	Time to flower	Biomass	Fruit no.	Herbivory1^a^	Herbivory2^a^
Site	2	**185.76** ***	**30.26** ***	**71.24** ***	**170.85** ***	**1229.24** ***	**859.70** ***	**34.92** ***	**468.73** ***
Population (Pop)	2	3.13	**5.83** **	3.82	3.93	**6.17** **	**6.26** **	3.16	1.22
Generation (Gen)	1	1.01	0.03	0.39	0.76	0.34	0.01	1.98	2.48
Cross type (Cross)	2	0.81	0.10	0.63	4.63	4.36	1.12	<0.01	1.01
Site × Pop	4	0.56	**4.29** **	1.92	0.21	**4.02** **	2.20	14.06	0.07
Site × Gen	2	1.69	2.30	2.58	1.88	1.24	1.22	**21.51** ***	4.61
Site × Cross	2	4.59	1.65	**5.22** *	1.27	3.06	**6.61** **	5.27	3.24
Pop × Gen	2	0.99	0.01	2.16	1.05	1.61	0.20	5.42	0.03
Pop × Cross	2	1.27	**6.84** **	**5.64** **	1.73	3.14	**5.97** **	2.69	3.13
Gen × Cross	2	0.03	0.16	0.20	0.66	0.29	0.28	0.94	1.83
Site × Pop × Gen	4	1.03	0.16	0.67	0.21	0.49	1.10	**19.32** ***	9.52
Site × Pop × Cross	4	0.41	1.15	2.02	1.30	1.49	2.63	9.56	**18.15** ***
Site × Gen × Cross	2	0.06	1.28	1.56	0.28	0.10	0.55	2.52	0.11
Pop × Gen × Cross	2	3.39	0.71	1.57	1.26	2.27	3.04	5.93	6.22
Site × Pop × Gen × Cross	4	1.96	2.53	1.20	1.75	0.58	3.18	**34.23** ***	2.50

a *Wald chi*‐square.

*, ** and *** indicate *P* ≤ .01, .005 and .001 respectively, significant after false discovery rates correction.

### 3.2 Testing local adaptation

The significant interaction between site and population may indicate local adaptation, which was further tested using local versus foreign population contrasts among the nonadmixed plants (intrapopulation crosses) at each site (Table 4). At the Tübingen common garden, the foreign plants had a higher biomass relative to the local Tübingen population, suggesting local maladaptation (indicated by “↓” in Table 4; Figure 1b,d). At the Potsdam site, although all plants were relatively small, the Potsdam population had a significantly higher biomass compared with foreign plants, suggesting local adaptation (indicated by “↑” in Table 4; Figure 1b,d). Interestingly, for total fruit production the pattern was reverse; the Potsdam population produced significantly less fruits than the foreign populations at the Potsdam site (Table 4, Figure 1a,c). Performance of the foreign and local plants at the Wageningen site was similar (Table 4; Figure 1).

**Table 4:** Local versus foreign population contrasts of the plant performance of intrapopulation crosses at each site, based on the same dataset and statistical model of Table 3. Table entries of traits are *F* values of the linear contrasts, *df* = 1, *N* = 60 per site. The data of the F1 and F2 generations were combined in these analyses

Trait	Potsdam	Tübingen	Wageningen
Height1	0.03	6.72↓	0.01
Height2	7.13↑**	26.62↓***	0.27
Height3	6.81↑**	11.24↓***	0.45
Time to Flowering	9.89**	0.39	0.75
Fruit number	60.57↓***	5.37↑	0.86
Total Biomass	3.57↑	7.38↓**	1.29

“↑” indicates the direction of contrasts (local > foreign, suggesting local adaptation); “↓” indicates local < foreign, suggesting local maladaptation.

** and *** indicate *P* ≤ .005 and .001 respectively, significant after false discovery rates correction.

### 3.3 Effects of admixture

There was no overall significant main effect of cross type on any of the measured plant traits (Table 3). However, the effect of cross type depended on experimental site for total fruit number and height3 (indicated by a significant site by cross type interaction, Table 3). At the Tübingen site, the admixed interregional offspring overall had more fruits and higher stems compared to the nonadmixed (intrapopulation crosses) offspring, or offspring of crosses between close‐by populations (interpopulation crosses) (Figure 1a,c, post hoc tests *p *≤* *.002). At the other sites, there was no significant overall effect of the cross type.

For fruit production and stem height, the effect of cross type also depended on plant population (Table 3). For the Potsdam population, fruit numbers and stem height overall increased with admixture, being lowest in the nonadmixed offspring and the highest in the interregional offspring (post hoc Tukey's tests, *p* ≤ .001). For the other two populations, the overall effect of cross type was less pronounced. We also did not find a main effect of cross type on herbivory. However, the three‐way interaction between cross type, site, and population was significant for herbivory2 (Table 3; Figure 2), suggesting that the effects of admixture on herbivore resistance depended both on plant genotype and on environment.

To test our hypothesis that admixture may negatively affect the performance of the home population, we performed post hoc tests between the nonadmixed (intrapopulation cross type) and the admixed offspring (interpopulation and interregion cross types) for each home population at their home site. Although not significant after correcting for multiple comparisons (FDR), admixture with distant populations (interregional crosses) had a positive effect on the biomass of the Tübingen population at its home Tübingen site (Figure 1b,d, *p* < .01). Biomass of the Potsdam and Wageningen populations was not affected by cross type at their home site. Fruit production increased with interregional admixture for the Potsdam population at the home site (Figure 1a,c, *p *<* *.0001). There was no effect of admixture on fruit production at the home site for the local Tübingen and Wageningen populations (Figure 1a,c, *p* > .05).

### 3.4 Performance of F1 versus F2 offspring

A main generation effect (difference between F1 and F2 generation) was not observed for any of the traits (Table 3). There was a significant interaction between population and generation for Herbivory1. In the Wageningen population, there was overall more herbivory in the F2 generation than the F1 generation in the first year (post hoc tests *P *=* *0.004), but there were no differences for the other two populations. The four‐way interaction between all four main factors including generation is also significant for herbivory in the first year of the experiment (Table 3).

## 4 DISCUSSION

Our reciprocal transplant study across three sites in the native range showed little evidence for local adaptation of European *L. salicaria*. However, depending on the environment, there was a positive effect of admixture on plant performance. In the following, we discuss these findings with respect to the initial questions.

### 4.1 Local adaptation

We found some home‐site advantage for the Potsdam population concerning plant growth but for not reproduction. The slightly shorter time to flowering of the Wageningen population in general may be an indication for adaptation to a shorter growing season in northwest Europe (Olsson & Agren, 2002). However, contrary to our expectations, there was no overall strong sign of local adaptation. There may be several reasons why we did not detect local adaptation in *L. salicaria* in this study. One reason for a potential lack of local adaptation could be a relatively small population size of the Tübingen and Wageningen populations (<500 flowering individuals, pers. observations J. Shi/K. Verhoeven). Small populations can have lower evolutionary potential to adapt to their environments relative to larger populations if genetic variation is low (Hill, 1982; Weber & Diggins, 1990). They may also suffer more from inbreeding depression, which could mask the benefits of local adaptation (Rius & Darling, 2014). Moreover, neutral genetic processes such as genetic drift may also occur more strongly in smaller populations and could lead to a loss of some advantageous alleles (Willi, Van Buskirk, Schmid, & Fischer, 2007). Possibly, continuous gene flow between *L. salicaria* populations, despite low population sizes, could hamper local adaptation (Slatkin, 1987). Using a common garden approach in three European regions, we likely tested regional adaptation (Weisshuhn, Prati, Fischer, & Auge, 2012), for example, adaptation to climatic conditions. The Wageningen site had milder winters and twice as much precipitation compared to the other two sites. A 4‐year large‐scale experiment in the invasive range of *L. salicaria* showed that there has been rapid adaptation to climatic gradients in North America (Colautti & Barrett, 2013). This was, however, over a much larger scale and larger climatic gradient than the West‐European study we present here, so perhaps if we would have included a broader range of European populations, we would have been able to detect adaptation to climate in the native range as well.

Intriguingly, the Potsdam population had a very high biomass at a foreign site, the Tübingen common garden. This high biomass may have been caused by two aspects: (1) escaping from some stressful local biotic and/or abiotic factors, such as low nutrient soils or local pathogens, which potentially restricted plant performance in Potsdam, (2) genomes of the Potsdam plants are preadapted to the Tübingen environment. For example, the climates of Tübingen and Potsdam are relatively similar (Table 1).

### 4.2 Costs of admixture: Outbreeding depression

We tested the effect of admixture on the three focal populations at our three transplant sites. If admixture dilutes local adaptation, we would expect a decrease in performance of the admixed offspring of the local plant genotypes at their home sites. Most previous studies that tested this assumption used a single field site (e.g., Cremieux et al., 2010; Keller et al., 2000). Because we found little evidence for local adaptation, our data do not provide a good basis for evaluating the cost of admixture that is associated with diluting locally adapted genomes. In the Potsdam region where we found some indications for local adaptation, there was a positive effect of admixture on fruit production for the home Potsdam population and no effect on plant growth. Possibly, the cost of dilution of local adaptation was counterbalanced or even overruled by the benefit of heterosis. For the other home populations, Tübingen and Wageningen, we also found no effect or a positive effect of admixture on performance at their home sites.

Theoretically, large geographical distances between plant provenances can lead to outbreeding depression via “hybrid breakdown” due to disruption of co‐adapted gene complexes (Hathaway et al., 2009; Wolfe, Blair, & Penna, 2007). Despite the fact that there is significant genetic differentiation between our European *L. salicaria* populations (Chun, Nason, & Moloney, 2009), we did not find a decrease in performance of our interregional crosses with large parental distances of 600 km, neither in the F1 nor in the F2 generation. This suggests that outbreeding depression via hybrid breakdown, at the scale of the regions included in this study, is unlikely to be of significant concern in mid‐European native *L. salicaria*. However, further rounds of crosses, that is, F3 or later generations, which we did not test here, may still reveal hybrid breakdown by disrupting co‐adapted gene complexes by further recombination.

### 4.3 Benefits of admixture: Heterosis

If there was a significant effect of admixture on the performance of our *L. salicaria* populations in the field, it was positive. This positive heterosis effect of admixture depended on both plant population and experimental site, which is in line with previous findings in other systems (Munaro, Eyherabide, D'Andrea, Cirilo, & Otegui, 2011). For the Tübingen plants at their home Tübingen site, a positive effect of admixture on shoot biomass was found both in the F1 and in the F2 generations. The Potsdam population overall benefitted from admixture. For both populations, this effect was strongest when plants were admixed with populations from distant regions, and less apparent or absent in admixture with close‐by populations. Genomes of geographically more distant populations are likely less similar than genomes of near‐by populations (isolation‐by‐distance), and therefore, the heterosis effect can be more pronounced. The observed heterosis could be due to the lift of inbreeding depression through admixture. For example, the Tübingen focal population size was very small, which could have led to inbreeding depression (Ellstrand & Elam, 1993; Young, Boyle, & Brown, 1996), although we did not test of the level of inbreeding in our populations. Our results suggest that mixing gene pools could be a potential tool to restore endangered small populations (Gellie et al., 2016). An alternative explanation for the boost of plant performance in admixed progeny particularly at the home site (Tübingen population) could be that some novel locally adapted genotypes have been created, for example, with higher resistance against herbivores or (soil) pathogens (Lavergne & Molofsky, 2007). However, we did not find a direct effect of cross type on herbivory in our experiment. There was no significant effect of admixture on the performance of the Wageningen population, neither at the home site, nor at the foreign sites.

The positive effect of admixture on some populations (mostly Tübingen) was not apparent in the Wageningen common garden. There, all plants performed more or less the same. However, some positive effects of admixture were found at the Tübingen and Potsdam sites. In Potsdam, all the plants were a lot smaller compared to the other sites, indicating that this might have been a more stressful environment, for example, a low nutrient soil or more drought. More stressful conditions may have led to a greater expression of inbreeding depression, and consequently also a more detectable heterosis effect. In general, the expression inbreeding depression is greater under stressful environments (Armbruster & Reed, 2005).

In conclusion, minor indications for local adaptation were only found in one of three *L. salicaria* populations. Dilution of local adaptation by admixture was not significant in our study, nor did we find indications of any hybrid breakdown. In the absence of local adaptation, admixture could lead to heterosis, and in our *L. salicaria* study the expression of heterosis depended on the environment. *L. salicaria* is highly invasive in North America (Colautti & Barrett, 2013), and there are indications that the invasive populations are admixed (Chun et al., 2009). Possibly, the increase in plant growth of *L. salicaria* in its invasive range is partly due to the heterosis effect of admixture (Verhoeven et al., 2011). Furthermore, our results suggest that in conservation, contrary to the current paradigm of only using local seed sources, admixture could be a tool to restore populations and/or increase population viability, especially in small inbred or maladapted populations (Gellie et al., 2016; Hufford & Mazer, 2003).

## CONFLICT OF INTEREST

None declared.

## AUTHOR CONTRIBUTIONS

JS, KJFV, and MM designed the experiment. JS, JJ, KT, KJFV, and MM performed the research. JS and MM analyzed the data and wrote the manuscript with contributions from all.
