# Supplementary material for: Costs and benefits of admixture between foreign genotypes and local populations in the field
Source: Ecol Evol. 2018 Mar 5;8(7):3675–84. doi: 10.1002/ece3.3946 (PMC5901173; doi:10.1002/ece3.3946)
Supplement: Supplementary file 1 [file ECE3-8-3675-s001.docx]

**APPENDIX**

Table S1. Average stem heights (SE) of F1 and F2 offspring of admixed (interpopulation and interregion crosses) and non-admixed (intrapopulation) offspring of three focal populations at three common gardens (site).

| **Site** | **Population** | **Cross type** | **Gen** | **Height1** | **Height2** | **Height3** |
| --- | --- | --- | --- | --- | --- | --- |
| Potsdam | Potsdam | Intrapopulation | F1 | 60.6 (2.7) | 64.8 (3.4) | 69.5 (3.2) |
|  |  |  | F2 | 70.8 (2.7) | 76.0 (4.7) | 79.5 (4.6) |
|  |  | Interpopulation | F1 | 63.63 (2.3) | 70.0 (6.0) | 75.3 (6.1) |
|  |  |  | F2 | 66.8 (3.1) | 67.7 (6.0) | 70.6 (5.9) |
|  |  | Interregion | F1 | 63.5 (5.9) | 66.2 (4.6) | 66.3 (4.2) |
|  |  |  | F2 | 74.0 (3.2) | 66.3 (11.0) | 77.4 (7.4) |
|  | Tuebingen | Intrapopulation | F1 | 63.5 (4.5) | 61.2 (5.4) | 62.9 (5.7) |
|  |  |  | F2 | 56.9 (2.6) | 52.1 (5.0) | 55.8 (4.7) |
|  |  | Interregion | F1 | 60.6 (2.3) | 63.5 (7.8) | 66.6 (7.7) |
|  |  |  | F2 | 75.1 (2.1) | 83.0 (4.1) | 85.9 (3.9) |
|  | Wageningen | Intrapopulation | F1 | 66.5 (3.7) | 64.3 (4.7) | 65.1 (4.7) |
|  |  |  | F2 | 74.0 (3.6) | 63.1 (4.9) | 65.9 (5.7) |
|  |  | Interregion | F1 | 73.1 (2.0) | 56.2 (3.1) | 59.0 (3.5) |
|  |  |  | F2 | 62.8 (2.8) | 64.5 (5.5) | 65.3 (5.3) |
| Tuebingen | Potsdam | Intrapopulation | F1 | 72.3 (4.2) | 79.8 (4.7) | 104.9 (7.9) |
|  |  |  | F2 | 69.8 (4.1) | 72.8 (5.4) | 102.3 (6.7) |
|  |  | Interregion | F1 | 77.0 (4.9) | 69.7 (7.7) | 97.7 (8.5) |
|  |  |  | F2 | 71.5 (3.4) | 73.6 (3.4) | 93.7 (3.9) |
|  | Tuebingen | Intrapopulation | F1 | 63.3 (3.2) | 49.7 (4.4) | 68.9 (3.8) |
|  |  |  | F2 | 63.9 (3.0) | 55.0 (4.2) | 87.0 (4.7) |
|  |  | Interpopulation | F1 | 68.9 (5.9) | 58.8 (4.9) | 86.5 (6.7) |
|  |  |  | F2 | 66.0 (8.6) | 51.4 (6.4) | 83.9 (8.6) |
|  |  | Interregion | F1 | 69.9 (5.1) | 59.0 (8.8) | 85.7 (12.1) |
|  |  |  | F2 | 72.8 (5.7) | 60.8 (5.0) | 98.0 (5.2) |
|  | Wageningen | Intrapopulation | F1 | 71.0 (4.6) | 66.0 (6.2) | 86.6 (7.3) |
|  |  |  | F2 | 68.8 (4.3) | 71.4 (4.3) | 81.1 (5.3) |
|  |  | Interregion | F1 | 78.4 (3.8) | 74.4 (3.2) | 96.0 (2.9) |
|  |  |  | F2 | 73.9 (4.7) | 69.8 (5.7) | 96.6 (5.1) |
| Wageningen | Potsdam | Interpopulation | F1 | 94.3 (4.0) | 58.1 (4.7) | 92.6 (5.8) |
|  |  |  | F2 | 102.6 (4.0) | 50.9 (4.3) | 92.3 (7.6) |
|  |  | Interregion | F1 | 94.1 (5.2) | 53.3 (3.7) | 96.6 (7.6) |
|  |  |  | F2 | 93.2 (3.1) | 45.7 (1.6) | 81.6 (4.7) |
|  | Tuebingen | Intrapopulation | F1 | 98.1 (4.5) | 52.4 (3.3) | 93.9 (4.7) |
|  |  |  | F2 | 95.2 (4.3) | 52.1 (2.5) | 96.7 (3.3) |
|  |  | Interregion | F1 | 93.3 (4.3) | 53.0 (4.7) | 87.4 (3.6) |
|  |  |  | F2 | 96.1 (3.3) | 51.0 (3.3) | 96.4 (7.5) |
|  | Wageningen | Intrapopulation | F1 | 99.3 (2.5) | 57.6 (2.9) | 103.6 (5.3) |
|  |  |  | F2 | 98.8 (3.1) | 53.2 (4.5) | 90.5 (4.3) |
|  |  | Interpopulation | F1 | 96.7 (3.2) | 52.9 (4.1) | 96.1 (6.4) |
|  |  |  | F2 | 100.6 (3.3) | 54.0 (5.4) | 96.0 (7.0) |
|  |  | Interregion | F1 | 93.0 (3.8) | 52.3 (1.9) | 95.8 (7.2) |
|  |  |  | F2 | 95.1 (4.2) | 52.4 (4.4) | 85.5 (6.2) |

Figure S1. The average monthly temperature (°C) and monthly precipitation (mm) in the regions of Tübingen, Potsdam and Wageningen
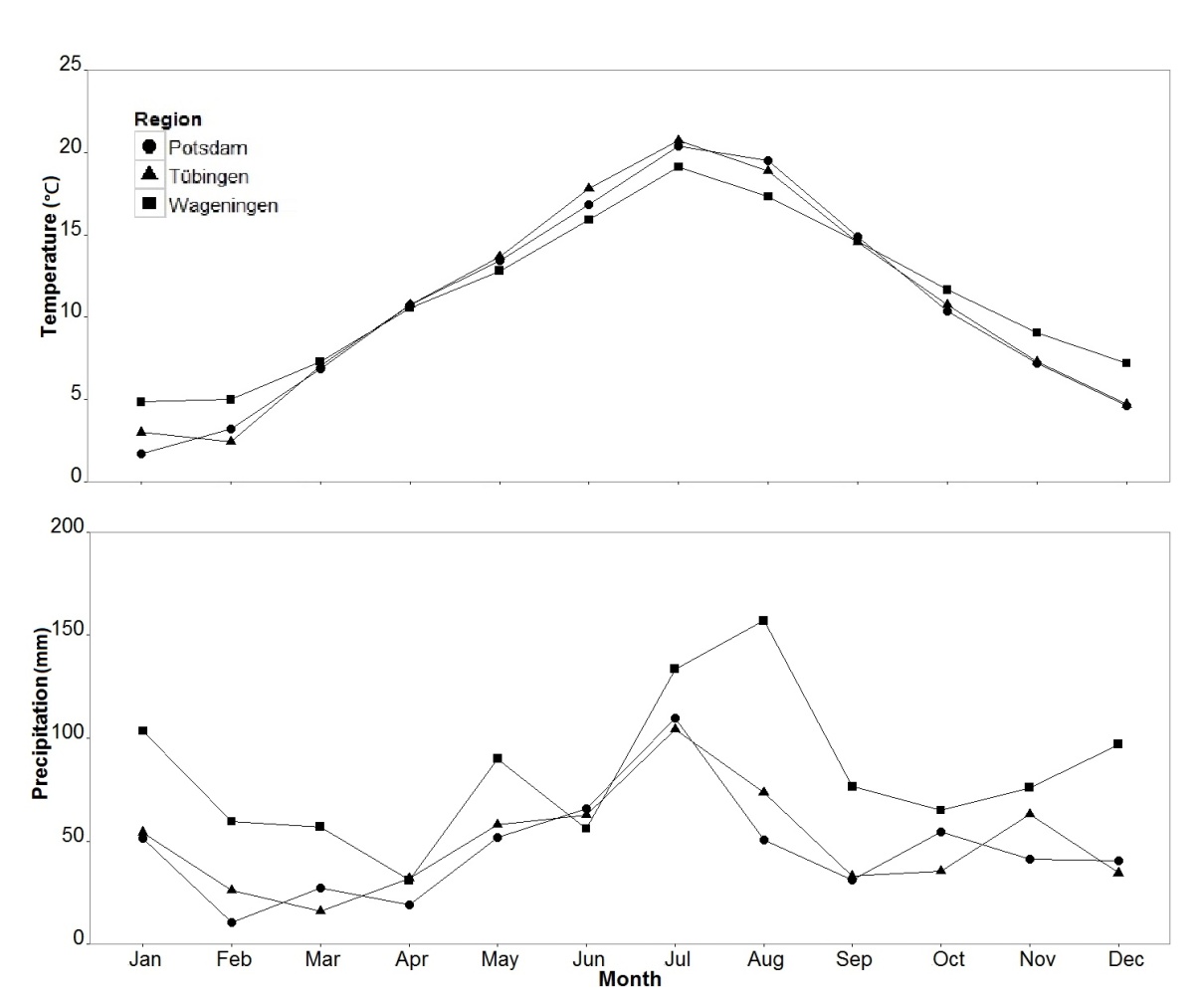
.
